# Well-being and its relationship with sports and physical activity of students during the coronavirus pandemic

**DOI:** 10.1007/s12662-021-00750-6

**Published:** 2021-09-22

**Authors:** Stefanie Pietsch, Stefanie Linder, Petra Jansen

**Affiliations:** Faculty of Human Sciences, University of Regensburg, Universitätsstr. 31, 93053 Regensburg, Germany

**Keywords:** Physical activity, Well-being, Optimism, Pessimism

## Abstract

The main goal of this correlational study is to examine the changes in the amount and type of physical activity of students of various study programs during the coronavirus pandemic. Furthermore, the motives for these changes as well as their relationship with general well-being are investigated. Therefore, 243 students (sport science, humanities, natural sciences) answered questions concerning (a) the amount and type of their physical activity before and during the pandemic, (b) their well-being and (c) their optimism/pessimism. The main result of the study is that the type and the motives for sports and physical activity changed for the study participants during the coronavirus pandemic: Team sports and swimming decreased, while individual sports and physical activity not associated with a sports club/business and with digital media increased. In this study the difference in sports and physical activity seemed to be related to well-being, especially for the students of sport science and humanities. To conclude the study indicated that a change occurred in the type of sports and physical activity as well as change in the motives for physical exercise in students during the pandemic.

A systematic engagement in sport (aiming for sport performance) as well as physical activity in general seems to have many positive effects, among them a beneficial influence on physical and mental health. Regular physical activity or sport with moderate intensity for instance can reduce the risk of cardiovascular diseases and depression (Lipowski & Zaleski, 2015; Mata et al., 2012; Meng & D’Archy, 2013). Physical activity can also lead to enhanced scholastic performance in children and more effective functioning across the lifespan (Hillmana, Kamijoab, & Scuddera, 2011). Current patterns and long-term trends reveal an overall trend of declining total physical activity and increasing sedentary behavior (Pate, Mitchell, Byun, & Dowda, 2011).

The coronavirus pandemic has changed the life of almost everyone. In this study we investigated the changes of the types of sport and physical activity, the motives for this change and the relationship with well-being in students during the pandemic. During the pandemic a further clear trend of decreasing physical activity was described. In France, Genin et al. (2021) reported a reduction of physical activity levels for children (42%), adolescents (58.7%), adults (36.4%) and older people (39.2%) during the confinement period. While in this study endurance activity decreased, strength and flexibility training at home increased. Those results could be confirmed by a meta-analysis demonstrating that most studies displayed a decrease in physical activity and an increase in sedentary behavior during specific lockdown times, which holds true for different populations (Stockwell et al., 2021).

However, it is known that participants who used digital platforms for physical activity had a higher moderate-to-vigorous intensity physical activity and muscle-strength training than nonusers (Parker et al., 2021). In Germany, leisure time sport and exercise decreased for 31% of Germans, while 27% retained and 6% even intensified their level. Thereby, younger persons more likely maintained their activity level compared to older ones by mainly practicing home-based workouts and outdoor endurance sports (Mutz & Gerke, 2021).

Motivation has been shown to be one of the crucial factors for steady physical activity. The self-determination theory (Deci & Ryan, 2002) provides a theoretical perspective explaining motivational issues for physical activity with regards to the processes of motivational development as well as the quality of motivation (Hagger & Chatzisarantis, 2007). Within the framework of this theory, the reasons for initiation and persistence of practicing physical activity and sports can be explained by specifying the social conditions, the nature and the function of motivation that affect motivational development and well-being. Self-determined motives for physical activity and sports are dependent on intrapersonal factors like basic psychological needs and interpersonal factors like autonomy supportive contexts (Wilson, Mack, & Grattan, 2008). Thereby the strong and important motives for sports and physical activity in adults differ depending on age, gender and the type of activity (Molanorouzi, Khoo, & Morris, 2015) and between active and inactive persons (Aaltonen, Rottensteiner, Kaprio, & Kujala, 2014). For active persons motives like physical fitness, social aspects, mastery, enjoyment, or the psychological state were significantly more important than for inactive persons (Aaltonen et al., 2014). Only regarding the motive of conforming to others’ expectations was more important for inactive persons compared to active persons. Even the motivation to practice different sport disciplines is multifaceted. Sports and physical activity like running, swimming, cycling or working out are often practiced because of health effects, while bodybuilding and working out in a gym are often exercised for a specific outward appearance. For sports like soccer, dancing or winter sports, pleasure is one of the main reasons (CBOS, 2013).

## Physical activity, sports and well-being in students during the coronavirus pandemic

Sport and physical activity were often restricted in the pandemic due to the closing of sports clubs and sports facilities as well as to the interdiction to train with other athletes during the lockdown periods. Furthermore, physical activity did play an important role in the handling of the pandemic. It was shown that patients with COVID-19, who were in general inactive, had a greater risk for hospitalization, intensive care unit admission and death (Sallis et al., 2021).

Given that the relationship between positive mental health and physical activity is well established (Bull et al., 2020) the maintenance of physical activity seemed to be crucial for the well-being during the pandemic. However, amount and way of practicing physical activity changed during the pandemic and especially during the lockdown periods: In a study conducted in Switzerland, the lockdown led, on the one hand, to longer times spent walking and in moderate physical activity and, on the other hand, to an increase in sedentary behavior during leisure time (Cheval et al., 2020). The questionnaires of this study were launched on the 30th of March and 4 weeks later. In the study by Brand, Timme, and Nosrat (2020) conducted during the first lockdown restriction with over 13,000 participants from 18 different countries, those participants who exercised almost every day during the pandemic had the best mood. This result was independent from the exercise behavior before the pandemic. The participants who reduced their exercise during the pandemic reported worse mood. This was confirmed in a sample of Italian citizens in a study during April 2020 (Maugeri et al., 2020). Also, from a study in Turkey, which started 8 weeks after the first case was mentioned in Turkey, it was concluded that physical activity should be included in guidelines as an integrative approach to pandemic management (Ozdemir et al., 2020).

Until now, physical activity of students during this pandemic in relation to their well-being has been already investigated in some studies: During the pandemic the amount and type of physical activity and sport decreased—with only 30% of university students achieving a “sufficient” level of activity (Gallo, Gallo, Young, Moritz, & Akison, 2020). In a study with students from the United States it was shown that the total minutes of physical activity were related to positive affect before and during the stay-at-home orders. The change of physical activity was positively associated with the change in psychological affect measured with the positive and negative affect scale. This relationship was neither moderated by stressful life events (Maher, Hevel, Reifsteck, & Drollette, 2021) nor by sleep quality, food insecurity or demographic factors. In a study with students from the Ukraine more students were involved in physical activity before the pandemic. In this study activity patterns were not related to the mood states. Students who suffered from anxiety and depression were almost two times less likely to engage in physical activity than their counterparts (Rogowska, Kuśnierz, & Bokszczanin, 2020). Wilson, Holland, Elliott, Duffey, and Bopp (2021) reported a significant decline in physical activity and an increased stress level during the pandemic in students from a northeastern university in the United States. Zhang, Zhang, Ma, and Di, (2020) concluded from a study with Chinese students that a suitable amount of daily physical activity and sleeping well might improve mental health. This was in line with the Chinese study by Lin et al. (2020) who reported that moderate-intensity physical activity was beneficial for reducing depression risk among college students. In all studies physical activity was measured with a physical activity questionnaire, but none of those studies registered the change of activity in different types of sport or physical activity.

Furthermore, to our knowledge, a change in the motives for doing sports and being physically active before and during the pandemic and a possible influence on future physical activity has not been reported so far. In addition to the main topic of the change of different types of sports and physical activity and their underlying motives, some demographic values should be registered while investigating the change in physical activity and their relationship with well-being. For example, concerning the gender of the participant the study by Bourion-Bedes, Tarquinio, Batt, Tarquinio, and Lebreuilly (2021) reported that to be female was one of the main predictors for the perceived stress during the pandemic. Also the existence of psychotic symptoms before the pandemic (Adorjan et al., 2021), the physical activity before the pandemic (but see Brand et al., 2020) as well as the study subject have to be considered. One might assume that sports students suffer more from a reduction in physical activity and sports, which in turn might be related to their well-being.

### Study goals

- The main goal of the study was to investigate whether the type and number of sports and physical activities has changed during the pandemic especially in German university students. It is expected that the sport types, which depend on sports facilities, sports clubs and team sports, will be reduced while sports and physical activity with digital media, physical activity that can be completed at home alone or individual outdoor sports and physical activity will increase.
- It is assumed that physical activity decreased during the pandemic in German university students. We reckon that physical activity can predict well-being, while taking into consideration—based on a literature review— other variables (gender, physical activity and the existence of psychotic symptoms, sports and physical activity difference, optimism and pessimism, living situation) and the study subject— a topic which has not been investigated before.
- Furthermore, it must be investigated whether the sport and physical activity and the motives for a change during the pandemic differs between students of different subjects. It is assumed that students of sport science are mostly affected, whereas no hypothesis could be formulated concerning students of humanities and natural sciences.

## Materials and methods

### Participants

In this study 223 students, 147 women (*M*_age_ = 22.62, *SD* = 2.03, between 19–31 years) and 76 men (*M*_*age*_ = 23.43, *SD* = 2.67, between 18–29 years) from different universities participated. To create a comprehensive and overreaching sample and to gain general results, data were gathered from students from throughout Germany. With a medium effect size *f* = 0.15, an alpha-level of *p* = 0.05, a power of 1–β = 0.95 and eight possible predictors, a power analysis for the linear regression resulted in a sample size of *n* = 160 participants. Participants were excluded from the study if they were not students of one of the three groups or did not complete the questionnaire correctly.

The students lived in the following living situations: 8.5% lived alone, 13.0% with a partner, 27.4% with the family, 39.9% in a shared apartment, 10.8% in a dormitory, and 0.4% chose “other” as an answer. Furthermore, 30.9% studied sport science, 32.7% humanities and 36.3% natural sciences. 5.3% said that they suffer from psychiatric syndromes, whereas 93.8% did not suffer from any such symptoms. 90.7% practiced sport and/or physical activity regularly for extended periods of time before the pandemic and 9.3% did not. The experiment was conducted according to the ethical guidelines of the Declaration of Helsinki. We communicated all considerations to the participants necessary to assess the question of ethical legitimacy of the study. In all, 496 participants opened the questionnaire once, 334 participants answered the questions on page 2 and 249 completed the questionnaire. From those 249 participants, 26 participants had to be excluded due to missing data or because they were not students.

## Measures

### Physical activity

#### Physical activity questionnaire.

Participants were asked which sports or physical activities they practiced regularly before the pandemic and since the beginning of the lockdown in November 2020. They were allowed to choose more than one of the following types of sport or physical activity using a yes/no answer: jogging, biking, swimming, ball sports (handball, soccer, volleyball, basketball, etc.), athletics, gymnastics, racket sport (tennis, badminton), fitness and strength training, fitness and strength training with digital media, fitness and strength training in a sports club/business, winter sport, hiking, climbing, yoga or others. The rate of the type of sports and physical activity from the students was recorded. Furthermore, they were asked whether they were restricted in their practice of their preferential type of sport and physical activity by the pandemic (1 = not at all, 2 = barely, 3 = partially, 4 = frequently, 5 = permanent), whether they found an alternative for their restricted type of sport or physical activity (1 = yes, 2 = no), whether they think that the change in their sportive activity will influence their future sport and physical activity after the pandemic (1 = not at all, 5 = very much), and their own well-being (1 = not at all, 5 = very much). Additionally, they were asked about their motives (relaxation, fitness, competition, health, appearance, social function, fun, boredom, loneliness, custom, incentive, duty) to practice sport or physical activity before the pandemic and during the lockdown (e.g., relaxation, to keep fit). For this, a 5-point Likert scale (1 = not at all–5 = very much) was used. Also we asked whether the participants suffered from psychotic symptoms (depression, anxiety, 1 = yes, 2 = no) before the lockdown and whether they were physically active before the pandemic (1 = yes, 2 = no).

#### IPAQ-SF (International Physical Activity Questionnaire Short Form, IPAQ Research Committee, 2005).

This questionnaire registers the physical activity in the last 7 days. It includes seven items: how many days participants spent in intensive and moderate exercise and while walking, and how many hours and minutes the specific intensity was achieved. In addition to the registration of physical activity in the last 7 days, participants were also asked to report their physical activity prior to the pandemic. The overall activity was measured in MET minutes per week by the sum: total MET minutes/week = Mod (METs · min · days) + Vig (METs · min · days), whereby moderate intensity = 4.0 METs and vigorous intensity = 8.0 METs. In addition, three groups of participants (with low, moderate, and vigorous physical activity [PA]) were designated according to the IPAQ guidelines. The reliability of the IPAQ-SF is 0.80 (Spearman’s rho), criterion validity is with 0.30 comparable to other studies with self-report validation (Craig et al., 2003).

### Well-being

#### WHO-5 Well-Being Index (Topp, Østergaard, Søndergaard, & Bech, 2015; for the German Version, Brähler, Muehlan, Albani, & Schmidt, 2007).

It measures current well-being during the previous 2 weeks. The five questions were positively phrased. An example for one of the questions is “I woke up feeling fresh and rested”. Participants responded using a six-point Likert scale (0 = at no time to 5 = all the time). In this study Cronbach’s alpha was 0.83.

#### Life Orientation Test–Revised (LOT-R; Scheier, Carver, & Bridges, 1994; German version by Glaesmer, Hoyer, Klotsche, & Herzberg, 2008).

The LOT‑R is a self-report instrument measuring optimism and pessimism. It measures individual differences in generalized optimism versus pessimism with 10 items (three positive optimism, three negative pessimism items, and four filler items) with a 5-point Likert-type scale ranging from 0 (strongly disagree) to 4 (strongly agree). Higher sum values indicate stronger optimism or pessimism (possible range 0–12). Cronbach’s alpha was 0.69 for optimism and 0.59 for pessimism (Scheier et al., 1994). In this study Cronbach’s alpha for the pessimism score was 0.66 and for the optimism scale 0.72, which was slightly higher than in the study of Scheier et al. (1994).

### Procedure

Participants completed the measures from 2 February 2021 to 18 February 2021 in the second wave of the pandemic in Germany. The online correlational study was implemented at SoSciSurvey. Participants were recruited by the authors through newsletters for students and social networks. After reading the study information, they accepted the consent form and completed the tests in the following order: demographic questionnaire, IPAQ, sports and physical activity before the pandemic and during the lockdown beginning in November 2020, WHO‑5, LOT‑R.

### Statistical analysis

First, sports activity and physical activity before the pandemic and since the November 2020 lockdown in Germany was analyzed using three univariate ANOVAs with the dependent variables: (a) restriction in the pandemic, (b) influence on future behavior, (c) influence on psychological well-being and the independent variable “study program”. Also, it was analyzed with the chi-square test if students have found an alternative to the restricted type of sport or physical activity done before. Second, the percentage of the specific types of sport or physical activity before the pandemic and during the lockdown was described. Chi-square tests were conducted to investigate whether there is a significant difference between the types of sport and physical activity practiced before and during the pandemic. Furthermore, it was investigated whether the difference in physical activity during the pandemic varies in students studying different subjects. An ANOVA with the difference score and the three groups of student subjects was conducted. Third, it was analyzed whether the motives for practicing sports and physical activity differ between the time before the pandemic and during the lockdown period.

Furthermore, the relationship between the difference in sports and physical activity measured with the IPAQ before and since the pandemic and the measurements of well-being (WHO‑5, optimism, pessimism) were computed with a Pearson correlation. Afterwards, a regression (method: Enter) of well-being (WHO-5) and the predictors: difference in sport and physical activity, optimism, pessimism, living situation, psychological symptoms, sports and physical activity before the pandemic, gender and study program was conducted.

## Results

### Sports activity and physical activity before and during the coronavirus pandemic

The results of the IPAQ indicated a change of activity behavior between the beginning of the pandemic and during the lockdown. Before the pandemic 17.49% of the participants displayed low activity, 20.62% moderate activity and 61.89% high activity. During the pandemic, registered 23.32% of the participants with low activity, 25.56% with moderate activity and 51.12% with high activity.

Because normal distribution was violated, the Kruskal–Wallis test was conducted to examine the differences in the restriction of the type of sport/physical activity between the different study groups. Significant differences were found (Χ^2^ = 7.71, *p* = 0.02, df = 2) among the students studying sports (Md = 128.57), humanities (Md = 100.61) and natural sciences (Md = 108.15). Post hoc Mann–Whitney tests were used to compare all pairs of groups. Students of sport science (Md *=* 90.35) feel significantly more restricted than students of humanities (Md = 63.14), *U*(N_SS_ = 69, N_HUM_ = 73) = 1908.00, z = −2.597, *p* = 0.009. There was no difference for students of natural sciences (Md = 68.92) compared to the other two groups. There was no significant effect for the different groups of students regarding influence on “future behavior” (Χ^2^ = 0.086, *p* = 0.958, df = 2) and no “influence on psychological well-being” (Χ^2^ = 1.212, *p* = 0.546, df = 2). In addition, 67% of the students reported that they have found and alternative to the type of sport/physical activity which they were not able to perform during the lockdown period, X^2^ (1, *N* = 194) = 22.45, *p* < 0.01.

Table 1 shows the frequency (%) of practicing a specific type of sport or physical activity before the pandemic and during the 2020 lockdown.

**Table 1:** Frequency (%) of practicing the respective type of sport or physical activity before and during the pandemic

Point of time	Sports/physical activity															
	Jog	Bik	Sw	Ball	Ath	Gy	Dan	Rac	Str1	Str2	Str3	Win	Hik	Cli	Yog	Ma
Before pandemic	40.4	63.2*	40.4*	49.8*	9.9 **	15.2	20.2**	20.6	47.1	13.5	44.4*	53.4**	51.6**	28.7**	17.5	9.89**
During lockdown	57.4*	41.3	2.7	4.0	3.1	2.2	10.3	4.5	52.5**	67.7*	8.5	15.2	43.5	9.9	39.5**	1.8

*Jog* jogging, *Bik* biking, *Sw* swimming, *Ball* ball sport, *Ath* athletics, *GY* gymnastics, *Rac* racket sport, *Str1* strength training on their own, *Str2* strength training with digital media, *Str3* strength training within a sports club/business, *Win* winter sport, *Hik* hiking, *Cli* climbing, *Yog* yoga, *Ma* martial arts

*X^2^: *p* < 0.05; ** X^2^: *p* ≤ 0.001; the higher value is marked

While sports and physical activity like swimming, team sports, dancing and martial arts decreased significantly, the time spent with individual sports and physical activity like strength training (on their own and with digital media) as well as yoga increased.

Furthermore, the univariate analysis of variance indicated that the difference score in the physical activity practiced before the pandemic and during the 2020 lockdown varies due to the study program, *F*(2, 183) = 3.980, *p* = 0.02, *η*_*p*_^*2*^ = 0.042. The difference was higher for the students of sport science (*M* = 1713.39, *SD* = 2693.47) compared to the students of humanities (*M* = 453.41, *SD* = 2217.05; *p* = 0.007). There was no statistical difference in sports and physical activity of students of natural sciences (*M* = 1225.08, *SD* = 2341.23) compared to the sports and physical activity of students of sport science (*p* = 0.275) and those of humanities (*p* = 0.071).

Table 2 shows the means given for the motives (1 = not at all, 5 = very much) to practice a type of sport or physical activity.

**Table 2:** Means (standard deviation) for the motives to practice a type of sport or physical activity before and during the pandemic

Point of time	Motives for sport/physical activity											
	Rel	Fit	Com	Hea	App	Soc	Fun	Bor	Lon	Cus	Inc	Dut
Before the pandemic	4.0 (1.1)	4.22 (0.094)	3.35** (1.47)	3.91 (0.98)	3.73 (1.14)	3.61** (1.38)	4.38** (0.94)	3.06 (1.31)	1.69 (1.00)	3.30** (1.47)	2.9* (1.33)	2.11** (1.48)
During the second lockdown	4.35** (1.04)	4.41* (1.03)	2.61 (1.49)	4.26** (0.97)	3.84 (1.2)	2.37 (1.55)	3.91 (1.2)	4.18** (1.17)	2.41** (1.41)	2.29 (1.4)	2.4 (1.34)	1.41 (0.98)

*Rel* relaxation, *Fit* fitness, *Com* competition, *Hea* health, *App* appearance, *Soc* social function, *Fun* fun, *Bor* boredom, *Lon* loneliness, *Cus* custom, *Inc* incentive, *Dut* duty

*Wilcoxon Z: *p* < 0.05; ** *Wilcoxon Z*: *p* ≤ 0.001; the higher value is marked

The results presented in Table 2 show significant differences between the motives to practice sports and physical activity. Sports and physical activity practiced according to the motives competition, social function, fun, custom and duty decreased significantly, while motives like relaxation fitness, health, boredom and loneliness gained in importance.

### Correlation between sports/physical activity and well-being

Table 3 shows the correlation between the difference in sports or physical activity (IPAQ Difference) and the variables of well-being measured by WHO‑5 and LOT‑R (optimism and pessimism).

**Table 3:** Correlational analysis with the difference score between the behavior before the pandemic and during the 2020 lockdown in sports/physical activity (IPAQ Difference) and the variables of well-being (WHO‑5, LOT-R: Optimism, Pessimism)

	IPAQ Difference	WHO‑5	Optimism	Pessimism
IPAQ Difference	1	r = −0.194, *p* = 0.008	r = 0.087, *p* = 0.242	r = 0.026, *p* = 0.729
WHO‑5	–	1	r = −0.409, *p* ≤ 0.001	r = −0.338, *p* ≤ 0.001
Optimism	–	–	1	r = −0.462, *p* ≤ 0.001
Pessimism	–	–	–	1

The results presented in Table 3 show that there is a correlation between the three well-being measurements and between the WHO‑5 and the difference in sports and physical activity. The correlation between the difference in IPAQ and the WHO‑5 were still significant for the students of sport science (r = −0.261, *p* = 0.034) and humanities (*r* = −0.271, *p* = 0.047) but not for those of natural science (*r* = −0.086, *p* = 0.503). Furthermore, the point-biserial correlation for all students between the existence of psychotic symptoms and well-being (WHO-5) and sports or physical activity before the pandemic and well-being showed two significant correlations: r = 0.239, *p* < 0.001 (psychotic symptoms, 1 = yes, 2 = no), and r = −0.232, *p* < 0.001 (sportive activity, 1 = yes, 2 = no).

### Prediction of well-being

The multiple regression results for the well-being scale indicated that 29.5% (*R* = 0.543) of the variance is explained by the predictors optimism, pessimism, difference of sports or physical activity, sports or physical activity before the pandemic and psychotic symptoms before the pandemic, *F*(8, 182) = 9.099, *p* < 0.001, Table 4.

**Table 4:** Regression of well-being with the predictors: optimism, pessimism, difference of sports or physical activity before and during the pandemic, gender, area of study, and psychotic and sports and physical activity before the pandemic

	Well-being					
Variable	b	SE	β	T	*p*	95% CI
Pessimism	−0.073	0.34	−0.156	−2.14	0.033	[−0.14; −0.01]
Optimism	0.121	0.34	0.267	3.61	< 0.001	[0.06; 0.19]
Sports/physical activity diff	< 0.001	< 0.001	−0.201	−3.03	0.003	[0.00; 0.00]
Living situation	0.095	0.057	0.109	1.67	0.097	[−0.02; 0.21]
Gender	0.083	0.133	0.041	0.620	0.536	[−0.18; 0.35]
Study subject	0.031	0.075	0.027	0.413	0.680	[−0.12; 0.18]
Psychotic symptom	0.755	0.335	0.150	2.256	0.025	[0.09; 1.42]
Sports/physical activity before	−1.146	0.334	−0.228	−3.427	0.001	[−1.81; −0.49]

*CI* confidence interval

The results presented in Table 4 report that well-being is predicted negatively by pessimism, and positively by optimism. Also, the higher the difference between sports and physical activity before and after the pandemic the lower the well-being. Furthermore, if psychotic symptoms appeared before the pandemic, well-being was lower. If the participants were physically active before the pandemic, their well-being was better during the pandemic.

## Discussion

First, our results indicated that the types of sport or physical activity as well as the motives for physical activity have changed during the pandemic. Second, the difference in sports and physical activity seemed to be related to well-being, especially for the students of sport science and humanities. Well-being could be predicted by the sports, physical activity, and psychotic symptoms before the beginning of the pandemic, the difference in sport or physical activity before the pandemic and during the lockdown period, and optimism and pessimism.

## Change of type of sport and physical activity during the coronavirus pandemic

According to the meta-analysis of Stockwell et al. (2021) the results of the IPAQ in this study demonstrated a decrease of students with a high activity level and an increase of students with low activity level. Concerning the type of sports and physical activity during the pandemic the amount of jogging, and fitness training without a sports club and with digital media as well as yoga increased, while the time spent with sports or physical activity like swimming, team sports, dancing and martial arts decreased. This result was not astonishing due to the restriction of some types of sports or physical activity, which could not be practiced in the pandemic anymore, like team sports or swimming. The importance of yoga as a preventive strategy in the pandemic was also verified by a review of Sawant, Zinjurke, and Binorkar (2021). Furthermore, more digitalized fitness programs were chosen, which is in line with a study of Parker et al. (2021) who reported higher physical activity of those people who practice sport or physical activity with the help of a digitized form.

The difference in the amount of sports and physical activity between before the pandemic and during the lockdown period was highest for sport science students and lowest for students of humanities, which could be explained due to the cancelled sport courses at university for sport students during the pandemic. In addition, students of sport science felt more restricted in their sporting exercise than students of humanities. This result gives a hint that the influence of sport and physical activity during the pandemic among students must be regarded as dependent on the study subject. Women generally prefer individual sports and sports or physical activity with strong esthetic elements, whereas men tend to participate in team sports and sports or physical activity that focus on strength or body contact (Colley, Berman, & Van Millingen, 2005). Therefore, students of humanities with a higher percentage of women could have preferred individual sports or physical activity like fitness, yoga or jogging already before the pandemic. In this case, the change in the type of sports or physical activity would not have been as severe as that for sport science students who depend on the quality of their sporting performance and the students of the natural sciences with a higher percentage of male students who mainly prefer sports or physical activities like team sports, martials arts or training in gyms. In our study, during the pandemic only 67% of the students found an alternative to types of sport or physical activity that they practiced before the pandemic. This is in line with the study of Mutz and Gerke (2021) who found less reduction in leisure time sports and physical activity during the pandemic in younger persons who practiced mainly home-based workouts and outdoor endurance sports already before the pandemic, while the persons who reduced their physical activity did not find adequate replacements for their sporting routines (Mutz & Gerke, 2021). Positively we found no significant effect to the influence on the future sports and physical activity behavior of the students.

### Change of motives for sports and physical activity during the coronavirus pandemic

Our results revealed significant differences between the motives for practicing sports and physical activity before and during the pandemic. Before the lockdown, motives like competition, social function, fun, custom and duty were important motives for students to participate in sports and physical activities. During the lockdown, the importance of these motives decreased significantly. The decrease of the motive duty could be referred to the cancelled courses of the students of sport science at university. In addition, competitions, mainly in team sports and sports with body contact were only allowed for professional athletes during the lockdown in Germany. The motive of social function became less important because sport and physical activity in groups was not possible during the pandemic. In contrast, motives like relaxation fitness, health, boredom and loneliness gained in importance. This is in agreement with the results of the study by Bösselmann et al. (2021). They found an association between physical activity, boredom and quarantine experience. Students with a higher physical activity level felt less bored and were less afraid of COVID-19.

### Physical activity and well-being among students

In line with previous studies (e.g., Brand et al., 2020) physical activity was related to positive emotions. Whereas in the study by Brand et al. (2020) mood was investigated, here well-being was related to physical activity as well as the concepts of optimism and pessimism, which is a more general view of life. Interestingly the relationship held true for the students of sport science and humanities but not for the natural sciences. This gives a hint that it is important to consider the subject of students when investigating the behavior of students during the pandemic. The results of the regression indicated that three factors are important for the well-being: sports and physical activity before and during the pandemic, the existence of psychotic symptoms, and the general orientation to life. Not only the behavior during the pandemic—but also prior to it—plays a crucial role. Javelle, Laborde, Hosang, Metcalfe, and Zimmer (2021) found similar results, showing that physical activity is a significant predictor of stress perception during the first lockdown. According to Wright, Williams, and Veldhuijzen van Zanten (2021) physical activity during the pandemic also seems to work against the negative effects of coronavirus fear on adolescents’ mental health and well-being. Besides changes in the type of sports and physical activity, it would be interesting not only to gather the time spent with sports and physical activity per week, but also the intensity of physical activity. Aghababa et al. (2021) found that while the intensity of physical activity of Iranian adults attending team sports decreased during the pandemic, the frequency of physical activity increased.

## Limitations

This study adds to former studies showing the relationship between physical activity and well-being but displayed empirical evidence in the change of different types of sports and physical activity (as assumed from a popular view) and the importance of differentiating among students from different subjects. However, the study is limited by the fact that it has not been recorded whether the students themselves or close friends were affected by the coronavirus. Also, data regarding socioeconomic status and sleep behavior are missing.

## Conclusion

This study contributes to raise awareness of the impact of the pandemic on sports and physical activity behavior of students. It can help students to reflect and improve the changes of their activity and the correlation to other parts of their everyday life and their psyche. Further studies could focus on the question of whether the changes in sports and physical activity during the pandemic were only temporary and students will return to their habitual activity behavior after the pandemic or whether the behavior in the field of sports and physical activity has changed permanently. In addition, the results could add to support sport possibilities for students during and after the pandemic.

## Declarations

For this article no studies with human participants or animals were performed by any of the authors. All studies performed were in accordance with the ethical standards indicated in each case.
